# Persisting *Cryptococcus* yeast species *Vishniacozyma victoriae* and *Cryptococcus neoformans* elicit unique airway inflammation in mice following repeated exposure

**DOI:** 10.3389/fcimb.2023.1067475

**Published:** 2023-02-14

**Authors:** Rachael E. Rush, Catherine B. Blackwood, Angela R. Lemons, Karen C. Dannemiller, Brett J. Green, Tara L. Croston

**Affiliations:** ^1^ Department of Microbiology, Immunology and Cell Biology, West Virginia University, Morgantown, WV, United States; ^2^ Health Effects Laboratory Division, National Institute for Occupational Safety and Health, Centers for Disease Control and Prevention, Morgantown, WV, United States; ^3^ Department of Civil, Environmental & Geodetic Engineering, College of Engineering, Ohio State University, Columbus, OH, United States; ^4^ Division of Environmental Health Sciences, College of Public Health, Ohio State University, Columbus, OH, United States

**Keywords:** yeast, fungi, inflammation, allergic disease, Vishniacozyma victoriae, Cryptococcus neoformans, exposure

## Abstract

**Background:**

Allergic airway disease (AAD) is a growing concern in industrialized nations and can be influenced by fungal exposures. Basidiomycota yeast species such as *Cryptococcus neoformans* are known to exacerbate allergic airway disease; however, recent indoor assessments have identified other Basidiomycota yeasts, including *Vishniacozyma victoriae* (syn. *Cryptococcus victoriae*), to be prevalent and potentially associated with asthma. Until now, the murine pulmonary immune response to repeated *V. victoriae* exposure was previously unexplored.

**Objective:**

This study aimed to compare the immunological impact of repeated pulmonary exposure to *Cryptococcus* yeasts.

**Methods:**

Mice were repeatedly exposed to an immunogenic dose of *C. neoformans* or *V. victoriae via* oropharyngeal aspiration. Bronchoalveolar lavage fluid (BALF) and lungs were collected to examine airway remodeling, inflammation, mucous production, cellular influx, and cytokine responses at 1 day and 21 days post final exposure. The responses to *C. neoformans* and *V. victoriae* were analyzed and compared.

**Results:**

Following repeated exposure, both *C. neoformans* and *V. victoriae* cells were still detectable in the lungs 21 days post final exposure. Repeated *C. neoformans* exposure initiated myeloid and lymphoid cellular infiltration into the lung that worsened over time, as well as an IL-4 and IL-5 response compared to PBS-exposed controls. In contrast, repeated *V. victoriae* exposure induced a strong CD4^+^ T cell-driven lymphoid response that started to resolve by 21 days post final exposure.

**Discussion:**

*C. neoformans* remained in the lungs and exacerbated the pulmonary immune responses as expected following repeated exposure. The persistence of *V. victoriae* in the lung and strong lymphoid response following repeated exposure were unexpected given its lack of reported involvement in AAD. Given the abundance in indoor environments and industrial utilization of *V. victoriae*, these results highlight the importance to investigate the impact of frequently detected fungal organisms on the pulmonary response following inhalational exposure. Moreover, it is important to continue to address the knowledge gap involving Basidiomycota yeasts and their impact on AAD.

## 1 Introduction

Allergic airway diseases including asthma, rhinitis, and similar respiratory morbidities have increased in incidence over time, specifically in industrialized nations such as the United States (Barnes, 2004; Bateman et al., 2008; Environment and Health Data Portal, 2020). These are complex inflammatory processes involving numerous cells and mediators that act directly or indirectly on the airway (Anderson, 2008). As a result, symptoms such as cough, chest tightness, shortness of breath, and wheezing may arise, which can impact quality of life and increase susceptibility to secondary pulmonary infections (Papi et al., 2018). This is particularly concerning during the COVID-19 pandemic, and it has been observed that individuals with severe asthma may be at increased risk for morbidity and mortality from COVID-19 infection (Howell et al., 2021).

Allergic asthma is the most diagnosed phenotype of asthma and is defined by sensitization to allergens, atopy, and is often triggered by environmental allergens (Eder et al., 2006). Individuals with allergic asthma are usually diagnosed early in life following symptom presentation and have increased total IgE and Th2 cytokine levels compared to non-allergic asthmatic counterparts (Schatz and Rosenwasser, 2014). Environmental allergens that are derived from microbial species present in indoor environments are often observed to influence allergic airway responses and lead to respiratory morbidities. Although pulmonary microbial exposures are well studied, most of the research has focused on the contribution of viruses and bacteria, and little is known about fungal exposures in general. For example, respiratory viruses such as rhinovirus frequently initiate asthma and are the most common trigger of asthma exacerbations (Jartti et al., 2020). Bacterial infections, such as *Mycoplasma* and *Streptococcus pneumoniae*, can impair mucous clearance, initiate goblet cell hyperplasia, and trigger IgE-mediated responses (Kraft, 2000) which exacerbate allergic airway diseases. However, it is becoming more evident that fungal microorganisms also contribute to allergic airway disease (Denning et al., 2014). A major caveat and reason the role of fungal species has not been recognized is the lack of standardized detection methods (Levetin et al., 2016; Green, 2018).

As Basidiomycota fungal species are not easily identifiable through traditional methods of fungal detection, these species have been largely overlooked, and as a result, vastly understudied. High throughput sequencing has changed the paradigm and consequently the identification of fungi in indoor environments contributing to human exposure (Alanio and Bretagne, 2014). Environmental assessments have emphasized the presence of Basidiomycota yeast species, specifically *Cryptococcus* (Adams et al., 2013; Dannemiller et al., 2014; Adams et al., 2015; Dannemiller et al., 2016). *Cryptococcus* yeasts include both pathogenic and nonpathogenic species. *Cryptococcus neoformans* is found in environments around the world, and is highly detected in pigeon droppings, and therefore, is commonly detected in urban areas (Levitz, 1991). *C. neoformans* is capable of growth at 37°C and has a rigid polysaccharide capsule that allows for the evasion of the immune system leading to cryptococcal infections primarily in immunocompromised individuals (Levitz, 1991; Idnurm and Lin, 2015; Campuzano and Wormley, 2018). *Cryptococcus victoriae* (since renamed *Vishniacozyma victoriae*) is highly prevalent in indoor environments (Pitkaranta et al., 2008; Dannemiller et al., 2016; Lutz et al., 2020; Rush et al., 2021). *Vishniacozyma victoriae* was isolated in the Antarctic (Montes et al., 1999) and has since been detected in indoor and outdoor environments worldwide (de Menezes et al., 2019). *V. victoriae* is also utilized for the control of post-harvest diseases of fruits in some semi-commercial settings in Argentina (Lutz et al., 2020). Unlike *C. neoformans*, *V. victoriae* grows optimally at lower temperatures (15°) but is known to tolerate a variety of environmental conditions. Additionally, *V. victoriae* does not have a polysaccharide capsule, which may contribute to its lack of pathogenicity. Studies have recently shown that *V. victoriae* produces a variety of compounds such as lipase, ergosterol, and linoleic acid (Rusinova-Videva et al., 2022). To date, *V. victoriae* research has not focused on the pulmonary immune response following exposure. Given the frequent and abundant detection in indoor environments, as well as its potential use in commercial settings, there is a crucial need to further examine the impact of exposure to *V. victoriae.*

Preliminary epidemiological-based studies suggest that *Cryptococcus* yeasts influence allergic airway diseases. Inhalation is the primary route of infection for *C. neoformans* (Campuzano and Wormley, 2018); however, pulmonary exposure to this yeast has also been shown to elicit allergic inflammation (Goldman and Huffnagle, 2009). One study using an ovalbumin model of allergic airway disease found that *C. neoformans* exposure exacerbated IgE responses and initiated eosinophil infiltration, goblet cell hyperplasia, and airway hyperreactivity (Goldman et al., 2006). Given the prevalence of *C. neoformans* in urban environments, it is important to note that individuals from these areas are also at a higher risk for asthma (Lin et al., 1999). Studies examining fungal exposure in health-based cohorts have indicated that low *Cryptococcus* diversity was highly associated with asthma development. Furthermore, previous studies found that *V. victoriae* concentrations in homes were inversely associated with asthma (Rush et al., 2021). Yet, a knowledge gap remains regarding the *in vivo* response to this frequently detected yeast species.

Given the inverse association of *V. victoriae* with asthma, as well as the differences between this species compared to *C. neoformans*, it is possible that exposure to *V. victoriae* influences pulmonary immune responses differently than *C. neoformans*. To better understand the impact exposure to *V. victoriae* may have on allergic airway disease, the pulmonary immune response to repeated exposure to each of these species was examined in parallel with *C. neoformans*. This was accomplished by repeatedly exposing mice to immunogenic doses of *V. victoriae* or *C. neoformans via* oropharyngeal aspiration, and then examining the physical changes, inflammation, mucous production, cellular influx, and cytokine response in the lungs of exposed mice at two different time points post-exposure. The immune responses following exposure to each yeast were characterized and compared to highlight the unique impacts of each exposure.

## 2 Materials and methods

### 2.1 Fungal cultivation and sample preparation

*Vishniacozyma victoriae* (ATCC MYA-305) was cultivated for exposures as previously described (Rush et al., 2021). Briefly, liquid cultures were grown in Potato Dextrose Broth (BD Biosciences, San Jose, CA) at 15°C. *C. neoformans* (ATCC 32045) was grown in liquid cultures in Yeast Mold Broth (BD Biosciences, San Jose, CA) at 25°C. Cells were collected during the logarithmic growth phase (approximately 48 hours post-inoculation for *V. victoriae* and 24 hours post-inoculation for *C. neoformans*), washed and diluted in phosphate buffered saline (PBS) at concentrations of 2x10^5^ cells/mL and 2x10^7^ cells/mL corresponding to 10^4^ and 10^6^ cells per 50 µL aspiration, respectively. Cells were diluted from fresh cultures each exposure day immediately prior to administration. Analysis of yeast growth kinetics at body temperature (37°C) compared to the optimal growth temperature was completed first by culturing *V. victoriae* from glycerol stock in 5 mL of PD broth at 15°C for 72 hours at 200 rpm. Flasks containing 100 mL of PD broth were inoculated with 100 µL of the liquid culture and incubated in a shaker incubator at either 15°C or 37°C at 150 rpm (n=2 per temperature). Optical density readings were obtained at various timepoints from 0 to 128 hours following inoculation using a SpectraMax M4 spectrophotometer (Molecular Devices, San Jose, CA). *C. neoformans* was cultured from glycerol stock in 5 mL of YM broth at 25°C for 72 hours at 200 rpm. Flasks containing 100 mL of YM broth were inoculated with 100 µL of the liquid culture and incubated in a shaker incubator at either 25°C or 37°C at 150 rpm (n=2 per temperature). Optical density readings at 600 nm were obtained at timepoints from 0 to 80 hours following inoculation.

### 2.2 Animal exposures

Randomly grouped 6- to 8-week-old female BALB/cJ mice (The Jackson Laboratory, Bar Harbor, ME) were used for all experiments. Preliminary studies demonstrated that female mice, in comparison to male mice, had a more severe allergic airway response to exposure, consistent with findings reported by Melgert et al. (2005). Mice included in the deposition study (n = 5 per group) were housed in the AAALAC International-accredited vivarium at the West Virginia University Health Science Center. Mice used in the repeated exposure and recovery study (n = 8-10 per group) were housed in the AAALAC International-accredited animal facility at the National Institute for Occupational Safety and Health (NIOSH). Mice received high-efficiency particulate absorbing (HEPA)-filtered air and were housed with 12-hour light/dark cycles and provided food and water *ad libitum*. All animal procedures were approved by the West Virginia University or CDC-Morgantown Animal Care and Use Committee.

**Figure 1:**
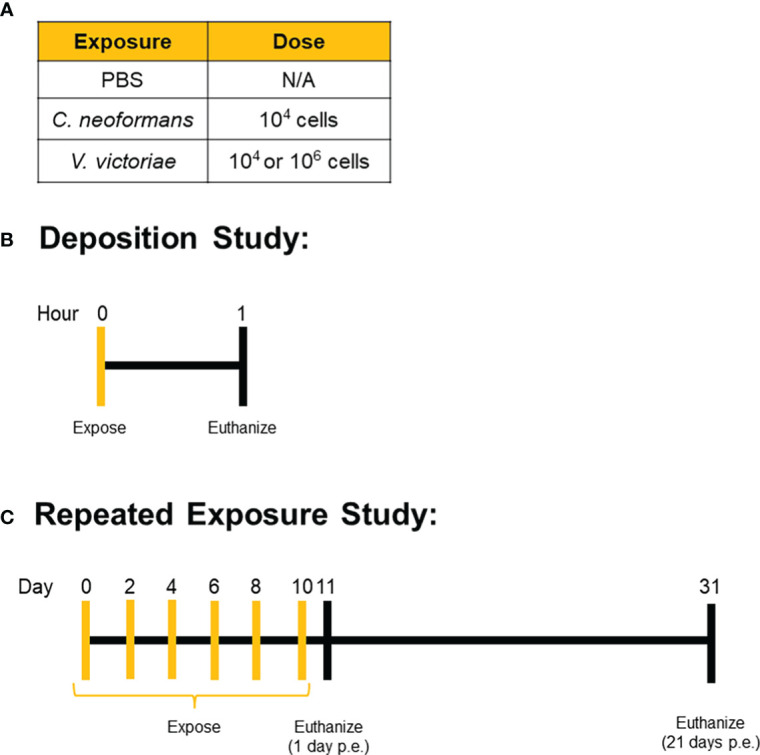
Exposure Paradigms. Mice were exposed to **(A)** set doses of either *C. neoformans*, *V. victoriae*, or PBS control *via* oropharyngeal aspiration. **(B)** Deposition was confirmed by exposing mice one time and then collecting tissue for analysis approximately 1 hour after the exposure. **(C)** For the repeated exposure study, mice were repeatedly exposed to the same dose of yeast or PBS control every other day for a total of six exposures, with tissue collection occurring either 1 day or 21 days after the final exposure.

Mice were exposed to yeast *via* oropharyngeal aspiration of a 50 µL yeast suspension prepared in PBS. For the deposition study, mice were anesthetized with isoflurane, exposed to one dose of yeast *via* oropharyngeal aspiration, and humanely euthanized approximately one hour later. Mice in the repeated exposure recovery study were anesthetized, exposed *via* oropharyngeal aspiration every other day for a total of six exposures, and then humanely euthanized 1 day or 21 days after the final exposure (**Figure 1**). All mice were humanely euthanized with an intraperitoneal injection of pentobarbital at 100-300 mg/kg body weight (Fatal Plus, Vortech Pharmaceuticals, Dearborn, MI). Serum, lung tissue, and bronchoalveolar lavage fluid (BALF) were collected following euthanasia for subsequent analyses.

### 2.3 Histopathological analysis

Capsule presence was examined by overloading murine lungs with approximately 10^8^ *C. neoformans* or *V. victoriae* cells, grown at 25°C and 15°C, respectively, which were then fixed with 10% neutral buffered formalin, paraffin embedded, sectioned and then stained with mucicarmine stain. Lung sections were visualized and images were captured at 100X magnification under oil immersion using an Echo Rebel microscope (Echo, San Diego, CA). Right lung lobes (n= 3/group) from repeatedly exposed mice were inflated with 10% paraformaldehyde fixative, tied off, and collected. Lung tissue was then embedded in paraffin, sectioned, and stained with hematoxylin and eosin (H&E), Periodic-acid Schiff (PAS), Grocott’s methenamine silver (GMS) stain for histopathological evaluation by iHisto services (Salem, MA). H&E-stained sections were examined to evaluate lung architecture, inflammation severity and location, and inflammatory cell types. PAS-stained sections were analyzed to identify changes in airway epithelium, specifically the proliferation of goblet cells and mucous production. GMS-stained sections were examined for yeast organisms and GMS^+^ cytoplasmic granules. Scores were determined for various parameters based on a standard qualitative toxicologic scoring scale (0 – none, 1 – minimal, 2 – mild, 3 – moderate, 4 – marked, and 5 – severe). Slides were imaged and analyzed by a blinded histopathologist, and scores and findings were reported (**Supplemental Table 1**). Lastly, embedded lung tissues were also stained with mucicarmine to identify the presence or absence of a capsule surrounding the yeast cells.

### 2.4 Flow cytometric analysis of lung tissue and bronchoalveolar lavage fluid

Bronchoalveolar lavage fluid (BALF) was collected from mice as previously described (Croston et al., 2020). Briefly, BALF cells were collected in 2 mL of PBS, washed, and immediately underwent flow cytometric staining. To analyze the remaining cells in the lung tissue, BALF-depleted lungs were then processed for flow cytometry. Following BALF collection, the lungs were washed with PBS, minced, and incubated in digestion buffer (5% fetal bovine serum, 1 mg/mL collagenase, 30 µg/mL DNAse Type IV in Dulbecco’s Modified Eagle Medium) for 30 minutes at 37°C on a shaker. Digested lung tissue was filtered (0.2 uM), washed, filtered, and centrifuged at 400 x g to isolate lung cells. Lung cells then underwent flow cytometric staining. Reagent information can be found in **Supplemental Table 2**.

Cell pellets from lung tissue were resuspended in 1 mL red blood cell lysis buffer for 5 min at room temperature, then washed in FACS buffer (4% fetal bovine serum + 2mM ethylenediaminetetraacetic acid in PBS) and centrifuged at 400 x g for 5 min. Next, lung and BALF cells were blocked in Fc Block (10% Rat Serum, 5% anti-mouse CD16/CD32 clone 2.4G2 antibody in PBS) for 5 min at room temperature. Cells were then incubated with the surface stain cocktail (**Supplemental Table 2**) for 25 min in the dark at 4°C. Following a wash, the cells were then fixed with Cytofix for 10 min at room temperature, washed, and resuspended in 250 ul of FACS buffer. Data were acquired using an LSR II flow cytometer (BD Biosciences). Cell counts were calculated utilizing Spherotech AccuCount Beads per the manufacturer’s instructions. Flow cytometric analysis was performed utilizing FlowJo version 10.6 (FlowJo, Becton, Dickinson and Company, NJ). Reagent information can be found in **Supplemental Table 1**.

### 2.5 Cytokine multiplex

Frozen lung tissue was homogenized utilizing the TissueLyser II system (Qiagen, Hilden, Germany). Briefly, lung tissues were placed in 2 mL Eppendorf tubes containing Tissue Protein Extraction Reagent (T-PER, Thermo Fisher Scientific, Waltham, MA) with a 5 mm metal bead (OPS Diagnostics, Lebanon, NJ) and lysed for three 30 second cycles at 4.5 Hz. Homogenized samples were centrifuged at 1,400 rpm for 10 min and the supernatant was collected and stored at -80°C for later use. Cytokine levels of murine IL-4, IL-5, IL-13, IL-33, and Eotaxin were assessed using a custom ProcartaPlex Multiplex Immunoassay (Invitrogen, Waltham, MA). The plate was prepared per the manufacturer’s instructions and run on a Luminex 200 system (Luminex, Austin, TX). In-software analysis was performed, and cytokine concentrations were extrapolated from averages of technical duplicates.

### 2.6 Statistics

Statistical analyses were performed using GraphPad Prism version 9 (GraphPad, San Diego, CA). Comparisons between exposures within the same time point were analyzed by ordinary one-way ANOVA with Tukey’s multiple-comparisons test. Comparisons of the same exposure across the two time points were analyzed by ordinary one-way ANOVA with Sidak’s multiple comparisons test unless otherwise noted. ROUT analysis was run to identify outliers that were removed. Heat maps were created utilizing the HeatMapper web tool (Wishart Research Group, Alverta, Canada).

## 3 Results

### 3.1 Yeast characterization and deposition in murine lungs following oropharyngeal aspiration

Phenotypic evaluation and growth curves were conducted to determine how each yeast species grow in different conditions. Mucicarmine stain is commonly utilized to stain polysaccharide capsules present in *C. neoformans* cells (Vance, 1961). To determine whether the cells of each yeast species had a capsule surrounding them, murine lungs were overloaded with *C. neoformans* or *V. victoriae* cells and stained with mucicarmine. As expected, *C. neoformans* cells stained a dark magenta color (**Supplemental Figure 1A**), indicating positive staining of the polysaccharide capsule. In contrast, *V. victoriae* cells did not stain the same indicative magenta color (**Supplemental Figure 1B**), suggesting the absence of a capsule surrounding these cells in the growth conditions utilized. Growth curves at optimal and body temperature were also conducted to determine if each yeast could grow at 37°C and potentially proliferate *in vivo.* As expected, *C. neoformans* was able to grow at 37°C (**Supplemental Figure 2A**, red) whereas *V. victoriae* was not (**Supplemental Figure 2B**, red). To validate that oropharyngeal aspiration was an appropriate method for pulmonary yeast exposures, mice were exposed to a single dose of yeast and lung tissue was collected and stained with GMS to identify deposited yeast cells. Following a single exposure, yeast cells were detected in the lung tissue of exposed mice specifically within free alveolar spaces, or in some cases, within alveolar macrophages (**Supplemental Figure 3**). The deposition was localized to the terminal bronchioles and alveolar duct junctions or within alveolar spaces (**Supplemental Figure 3**). This confirmed that the oropharyngeal aspiration effectively delivered yeast into the lungs of mice.

### 3.2 *C. neoformans* and *V. victoriae* induced airway inflammation

**Figure 2:**
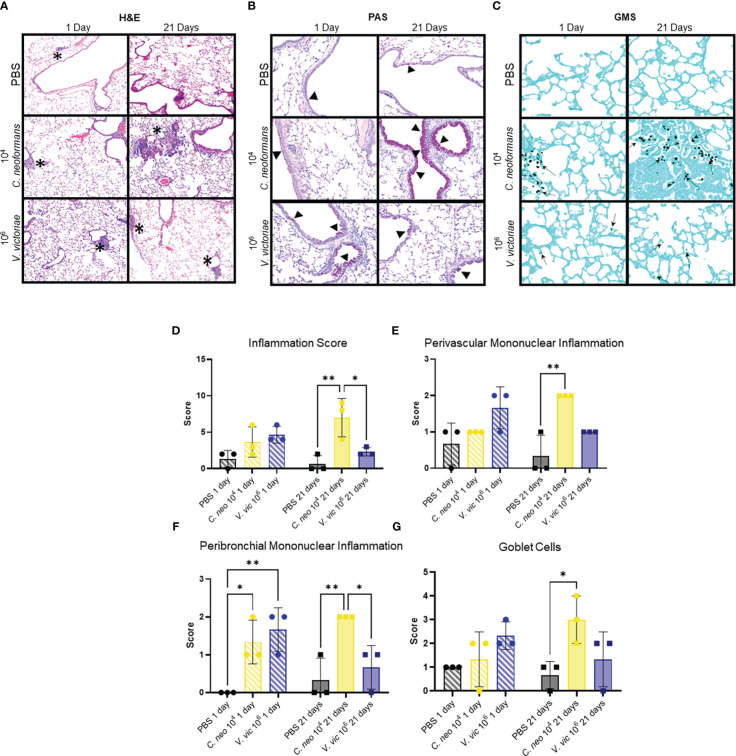
*C. neoformans*-induced inflammation worsens over time, whereas *V. victoriae*-induced inflammation resolves. **(A–C)** Representative micrographs of stained lung sections from mice one day (left) or 21 days (right) following repeated exposure to PBS (top), 10^4^ *C. neoformans* (middle), or 10^6^ *V. victoriae* cells (bottom). **(A)** H&E stain, 100x magnification. **(B)** PAS-stain, 200x magnification. **(C)** GMS-stain, 400x magnification. **(D)** Inflammation, **(E)** Perivascular Mononuclear Inflammation, **(F)** Peribronchial Mononuclear Inflammation and **(G)** Goblet Cells histology scores of lungs following repeated exposure to PBS (black), 10^4^ *C. neoformans* cells (yellow) or 10^6^ *V. victoriae* cells (blue) at 1 day post final exposure (circles, crossed bars, left) or 21 days post final exposure (squares, solid bars, right). n=3 per group, *P<0.05, **P < 0.01. P values were determined by ordinary one-way ANOVA with Tukey’s multiple comparisons test comparing each exposure together for the same timepoint.

Given the frequent detection of yeasts in indoor and outdoor environments, mice were repeatedly exposed to *C. neoformans* and *V. victoriae*, or PBS, to mimic a sustained pulmonary exposure over time (Levitz, 1991; Rush et al., 2021). One day following repeated PBS aspiration, mild inflammation, and mucous production were observed in the lungs of control mice (**Figures 2A, B**). Furthermore, the minor inflammatory response persisted 21-days post final exposure (**Figures 2A, B**). No GMS-positive cells were detected in the lungs at either time point following PBS exposure (**Figure 2C**). Along with mild mucous production, moderate perivascular and peribronchiolar inflammation was observed one day following repeated 10^4^ *C. neoformans* exposure (**Figures 2A, B**). *C. neoformans* cells were also detected in the lung tissue as free cells and within alveolar histiocytes as shown by GMS and mucicarmine staining (**Figure 2C**; **Supplementary Figure 4A**). By 21-days post final *C. neoformans* exposure, the inflammation persisted, and granuloma formation was observed (**Figure 2A**). There was also moderate to marked mucous production and goblet cell hyperplasia present at 21 days post final exposure (**Figure 2B**). The yeast cells were still detectable at this time point, sometimes within lesions (**Figure 2C**; **Supplementary Figure 4B**), and increased in number as evidenced by histological counting. As an inflammatory response was evident following 10^4^ *C. neoformans* exposure and for concerns of morbidity, a higher dose of *C. neoformans* was not considered. Together, these findings suggest that *C. neoformans* cells persist in the lungs of exposed mice and initiated a strong pulmonary inflammatory response over time.

Following repeated exposure to 10^6^ V. victoriae cells, mice exhibited perivascular and peribronchiolar inflammation including nodular inflammation (**Figure 2A**). Moderate mucous production and goblet cell hyperplasia were present, and GMS-positive histiocytes were detected at 1-day post final exposure (**Figures 2B, C**). No mucicarmine-positive stained yeasts were visualized following *V. victoriae* exposure (data not shown). By 21 days post final *V. victoriae* exposure (10^6^ cells), the inflammation and mucous production had started to resolve (**Figures 2A, B**). Still, GMS-positive histiocytes were detected in exposed mice (**Figure 2C**). In comparison to exposure to 10^4^ *C. neoformans* cells, results indicated that *V. victoriae* cells also persist within histiocytes in the lung over time; however, the observed pulmonary inflammation started to resolve by 21 days post final exposure unlike with *C. neoformans*. Furthermore, mice did not lose significant weight and appeared healthy throughout the studies suggesting that an immunogenic response, but not a pathogenic infection, was achieved utilizing these doses and timeframes as intended.

Exposure to each of the yeasts elicited a mononuclear inflammatory response inclusive of lymphocytes and plasma cells (**Figure 2A**). *C. neoformans* exposure also elicited a mixed response including neutrophils and granuloma formation with macrophages and multinucleated giant cells (**Figure 2A**; **Supplementary Table 2**). Scores for total, perivascular mononuclear, and peribronchial mononuclear inflammation were provided by a blinded veterinary pathologist and results indicated that repeated exposure to 10^4^ *C. neoformans* and 10^6^ *V. victoriae* cells induced a similar pulmonary inflammatory response (**Figures 2D–F**). Furthermore, these exposure groups also had similar goblet cell scores (**Figure 2G**). Only *C. neoformans*-exposed mice had detectable free yeast cells, whereas all *V. victoriae*-exposed mice had GMS-positive histocytes (**Supplementary Table 2**). These results suggest that *V. victoriae* had been phagocytosed by immune cells.

**Figure 3:**
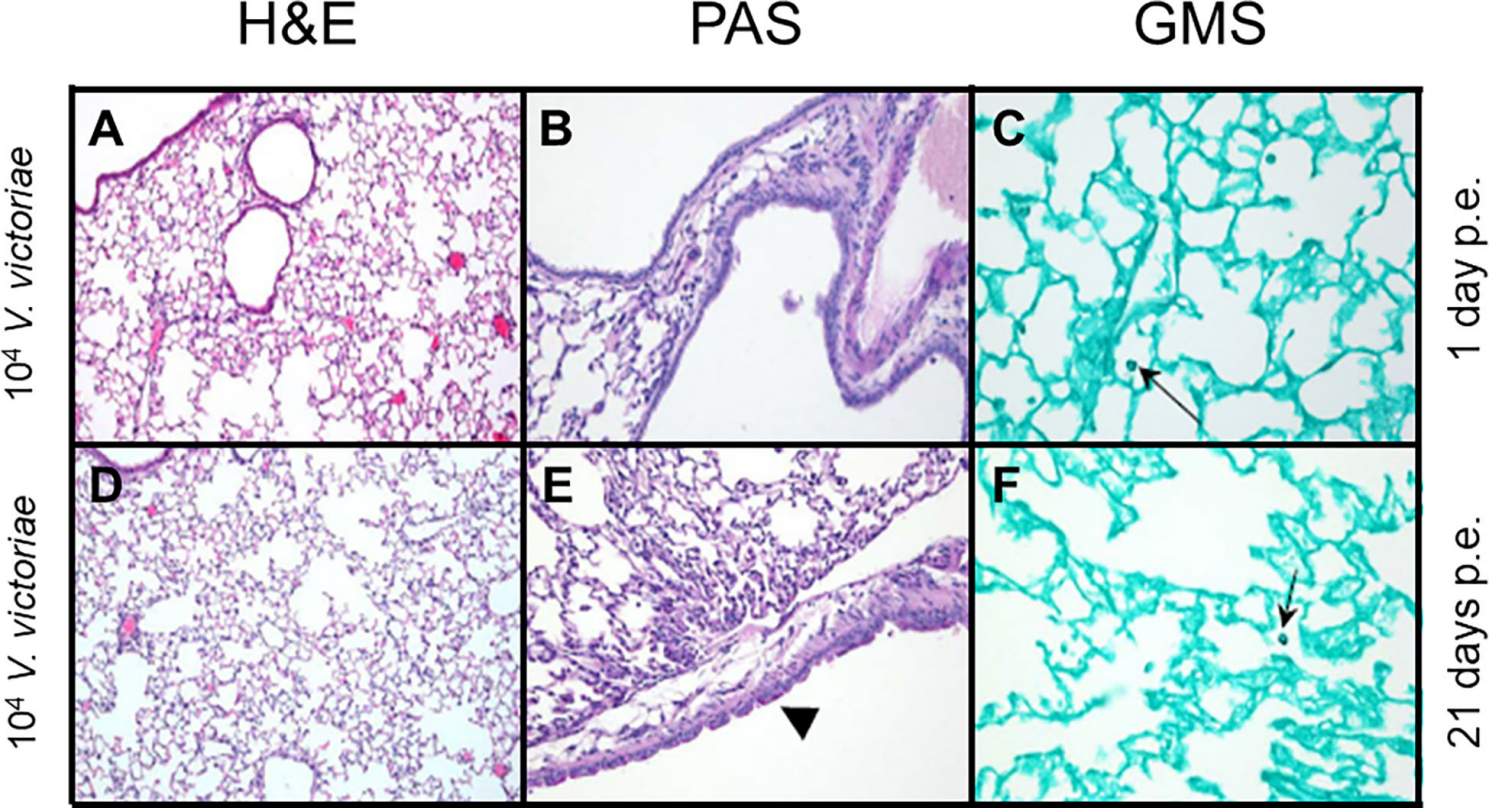
Inflammation, airway changes, and yeast infiltration in lungs of mice after repeated exposure to 10^4^ *V. victoriae* cells. Representative micrographs of stained sections of lung from mice 1 day (top) and 21 days (bottom) following repeated exposure 10^4^ *V. victoriae* cells. Goblet cells are by arrowheads, and yeast cells by arrows. **(A, D)** H&E stain, 100x magnification. **(B, E)** PAS-stain, 200x magnification. **(C, F)** GMS-stain, 400x magnification.

Following repeated exposure to 10^4^ *V. victoriae* cells, the same dose as *C. neoformans*, mice exhibited very little to no inflammation, mucous production, or goblet cell hyperplasia one day post final exposure (**Figures 3A, B**; **Supplementary Table 2**); however, GMS-positive histiocytes were present in the lung tissue (**Figure 3C**; **Supplemental Table 2**). By 21 days post final *V. victoriae* exposure (10^4^ cells), little to no inflammation or mucous production remained (**Figures 3D, E**; **Supplementary Table 2**). Surprisingly, GMS-positive histiocytes were still detected in the lung (**Figure 3F**; **Supplementary Table 2**). Given that exposure to 10^4^ *V. victoriae* cells did not elicit an immune response, a higher, immunogenic dose of 10^6^ *V. victoriae* cells was used to further characterize the pulmonary immune response to *V. victoriae*.

### 3.3 *C. neoformans* and *V. victoriae* impacts on myeloid populations in BALF

**Figure 4:**
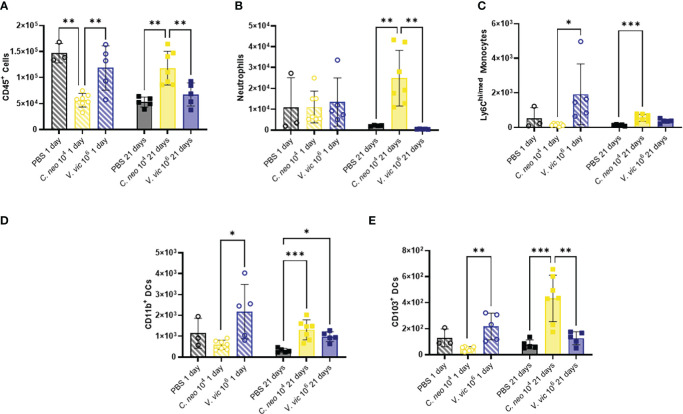
*C. neoformans* exposure has a stronger impact on myeloid populations in the BALF over time compared to *V. victoriae*. Cell quantifications (numbers) in BALF following repeated exposure to PBS (black), 10^4^ *C. neoformans* cells (yellow) or 10^6^ *V. victoriae* cells (blue) at 1 day post final exposure (circles, crossed bars, left) or 21 days post final exposure (squares, solid bars, right). **(A)** CD45^+^ Cells, **(B)** Neutrophils, **(C)** Ly6C^hi/med^ Monocytes, **(D)** CD11b^+^ Dendritic Cells, and **(E)** CD103^+^ Dendritic Cells. n=3-7 per group, *P<0.05, **P < 0.01, ***P < 0.001. P values were determined by ordinary one-way ANOVA with Tukey’s multiple comparisons test comparing each exposure together for the same timepoint.

*Cryptococcus neoformans* has a polysaccharide capsule that protects the cells from phagocytosis (Yang et al., 2017), whereas *V. victoriae* does not; however, beta-glucans present in both yeast cell walls are known to stimulate innate immune cells (Goodridge et al., 2009). Given this, the impact of repeated exposure to each yeast on the BALF myeloid cell population was examined (**Supplementary Figure 5A**). Neither yeast significantly increased myeloid populations of interest in the BALF by one day post final exposure compared to PBS exposed mice (**Figure 4**). Repeated exposure to 10^6^ *V. victoriae* cells increased Ly6C^hi/med^ monocytes, CD11b^+^ DCs, and CD103^+^ DCs by 1-day post final exposure compared to *C. neoformans* exposure (**Figures 4C–E**).

Repeated *C. neoformans* exposure increased total CD45^+^ cells, neutrophils, Ly6C^hi/med^ monocytes, CD11b^+^ DCs and CD103^+^ DCs by 21 days post final exposure (**Figure 4**) compared to PBS control. Following repeated exposure to 10^6^ *V. victoriae* cells, only a significant increase in CD11b^+^ DCs remained after 21 days post final exposure (**Figure 4D**) compared to PBS control. Surprisingly, neither yeast exposure increased eosinophil nor macrophage quantifications in the BALF (**Supplementary Figure 6**). There were no significant differences in cellular populations in the BAL-depleted lungs that was not evident in the BALF (**Supplementary Figures 7**–**9**). Taken together, these findings indicate that repeated exposure to 10^6^ *V. victoriae* cells elicited a myeloid-driven immune response at 1-day post final exposure that was largely resolved by 21 days post final exposure, whereas repeated exposure to 10^4^ *C. neoformans* cells had a delayed impact on myeloid immune responses.

### 3.4 Lymphoid populations in BALF following yeast exposures

**Figure 5:**
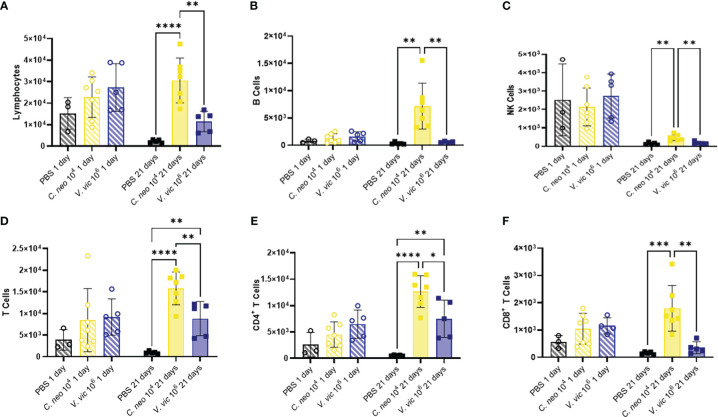
Lymphoid populations are elevated in the BALF 21 days following repeated yeast exposure. Cell populations in BALF following repeated exposure to PBS (black), 10^4^ *C. neoformans* cells (yellow) or 10^6^ *V. victoriae* cells (blue) at 1 day post final exposure (circles, crossed bars, left) or 21 days post final exposure (squares, solid bars, right). Cells = cell number. **(A)** Lymphocytes, **(B)** B Cells, **(C)** NK Cells, **(D)** T Cells, **(E)** CD4^+^ T Cells, and **(F)** CD8^+^ T Cells. n=3-7 per group, *P<0.05, **P < 0.01, ***P < 0.001, ****P < 0.0001. P values were determined by ordinary one-way ANOVA with Tukey’s multiple comparisons test comparing each exposure together for the same timepoint.

Lymphocytes are known to play an important role in the immune response against *C. neoformans* (Muth and Murphy, 1995; He et al., 2017); therefore, lymphocytic cell populations were characterized (**Supplementary Figure 5B**). There were no significant differences in lymphoid cell populations in the BALF 1-day post final exposure between yeast-exposed mice and PBS controls (**Figure 5**). BALF lymphocyte populations increased 21 days following repeated yeast exposure. Specifically, repeated exposure to 10^4^ *C. neoformans* cells resulted in increased total lymphocytes, B cells, Natural Killer cells, total T cells, CD4^+^ T cells, and CD8^+^ T cells compared to PBS exposed controls (**Figure 5**). In contrast, only total T cells and CD4^+^ T cells were increased by 21 days post final exposure following repeated exposure to the 10^6^ cell dose of *V. victoriae* (**Figures 5D, E**). These findings suggest that repeated exposure to both yeast species elicited a strong lymphoid response, specifically CD4^+^ T cells, by 21 days post final exposure.

**Figure 6:**
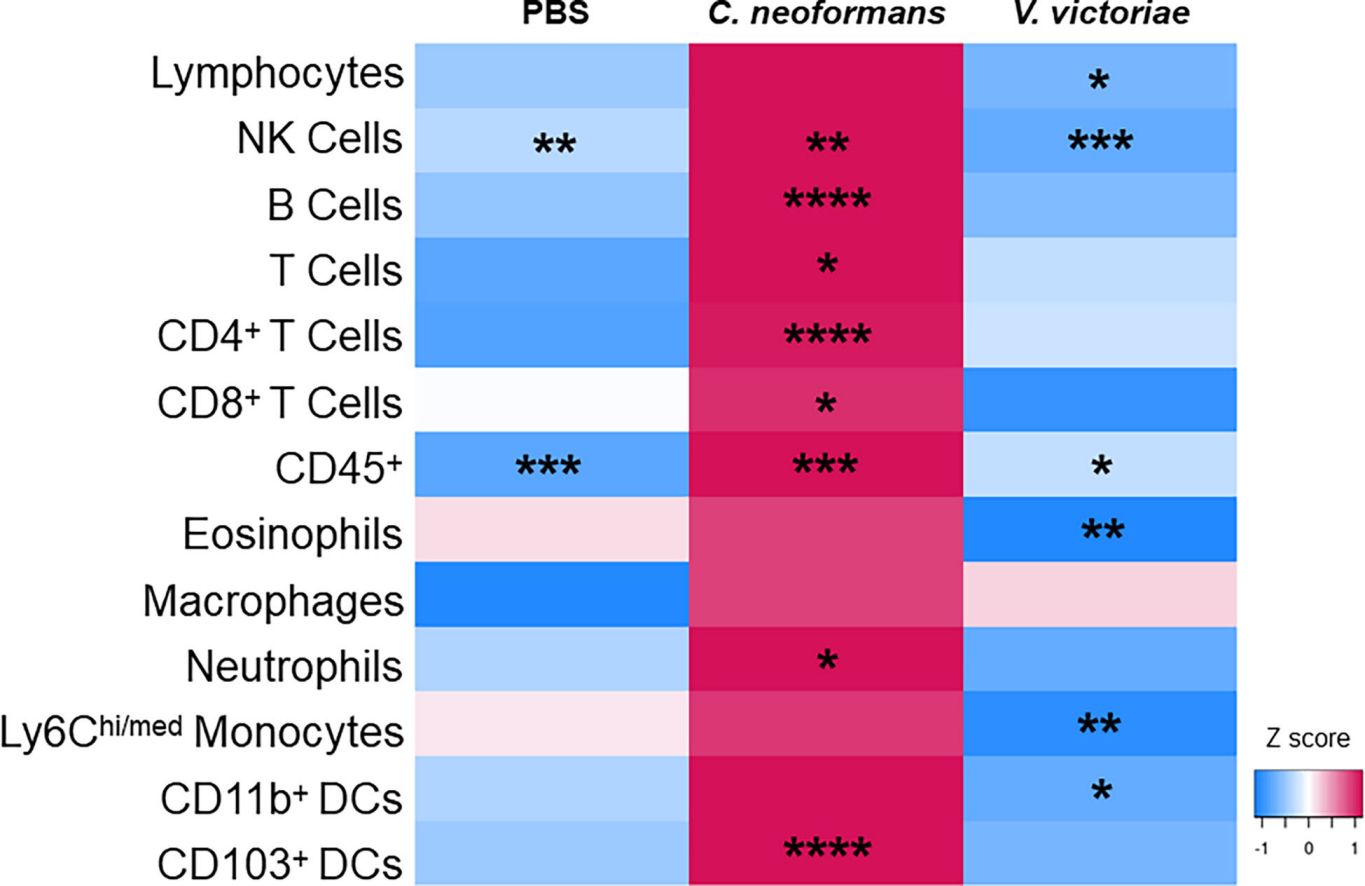
Repeated *C. neoformans* exposure continues to increase cell populations in the BALF at 21 days post final exposure. Mean cell quantifications in BALF following repeated exposure to PBS, 10^4^ *C. neoformans* cells or 10^6^ *V. victoriae* cells at 1 day post final exposure compared to 21 days post final exposure. n=3-7 per group. Values used for this heatmap were obtained by calculating the average quantities at 21 days post final exposure and subtracting the average quantities at 1 day post final exposure. A positive Z score (pink) indicates that the quantification of cells increased at 21 days post final exposure compared to 1 day post final exposure. Negative Z scores (blue) indicate that the cell quantifications decreased by 21 days post final exposure compared to 1 day post final exposure. Asterisks overlaying the heatmap indicate P values comparing the quantifications of each time point for the same exposure. *P<0.05, **P < 0.01, ***P < 0.001, ****P < 0.0001. P values were determined by ordinary one-way ANOVA with Sidak’s multiple comparisons test comparing the same exposure at 1 day post final exposure and 21 days post final exposure.

Research has shown that immune cell infiltration worsens over time following pulmonary exposure to *C. neoformans* (Goldman et al., 2006); however, no studies exist examining the potential exacerbation of, or recovery from, the cellular response following pulmonary exposure to environmental yeasts. Therefore, the differences in concentrations of cell populations between one-day and 21 days post final yeast exposure were examined. Repeated exposure to 10^4^ *C. neoformans* cells continued to cause an increase in cell populations in the BALF after the final exposure. In contrast, the cellular impacts following repeated exposure to 10^6^ *V. victoriae* cells start to decrease over time after the final exposure (**Figure 6**).

### 3.5 Lung cytokine responses following repeated yeast exposures

**Figure 7:**
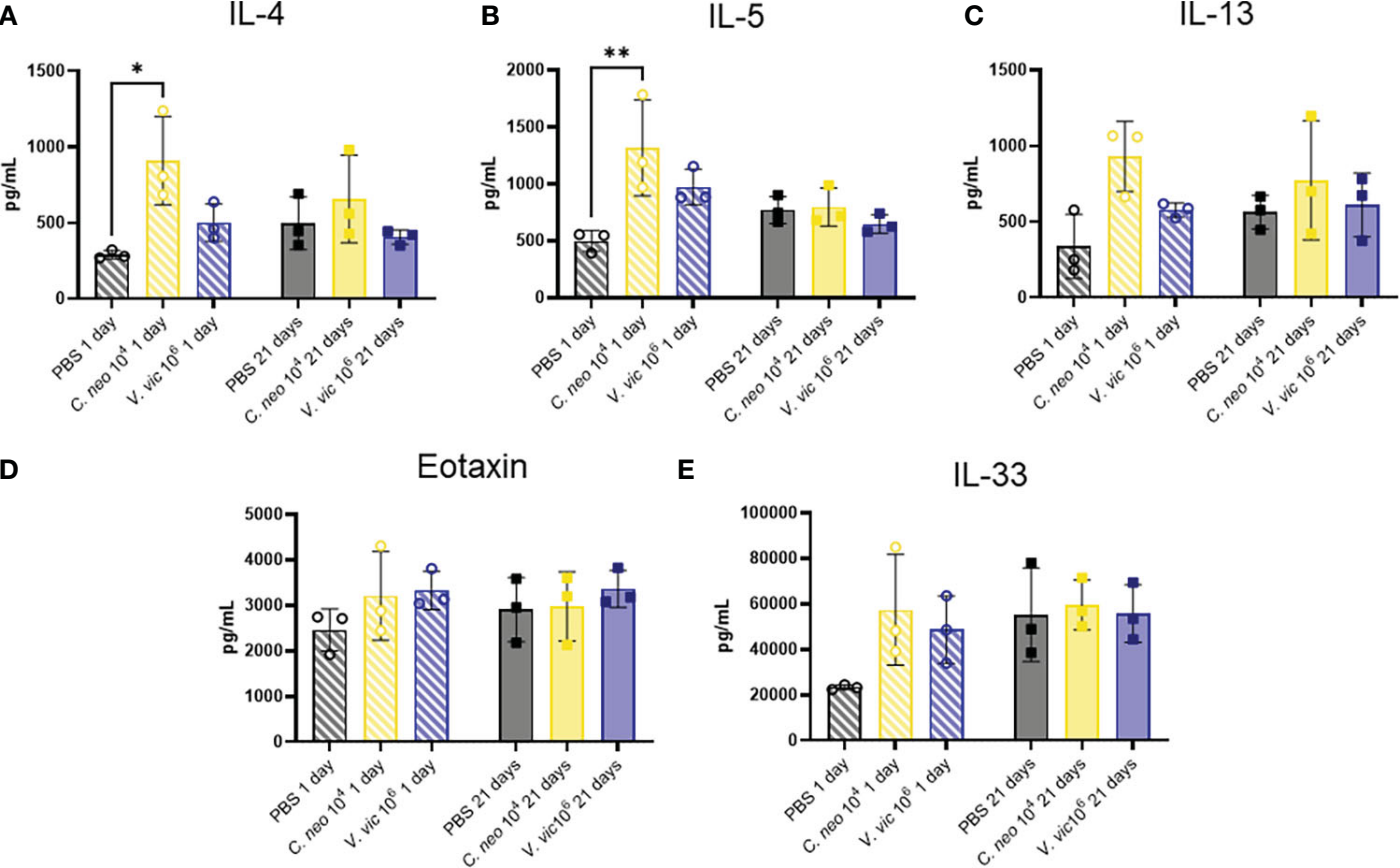
*C. neoformans* increases IL-4 and IL-5 in lung one day following repeated exposure. Cytokine concentrations in lung homogenate were determined *via* Luminex after repeated exposure to PBS (black), 10^4^ *C. neoformans* cells (yellow) or 10^6^ *V. victoriae* cells (blue) at 1 day post final exposure (circles, crossed bars, left) or 21 days post final exposure (squares, solid bars, right). **(A)** IL-4, **(B)** IL-5, **(C)** IL-13, **(D)** Eotaxin (CCL11), and **(E)** IL-33. n=3 per group, *P < 0.05, **P < 0.01. P values were determined by ordinary one-way ANOVA with Tukey’s multiple comparisons test comparing each exposure together for the same timepoint.

The Th2 cytokines IL-4, IL-5, and IL-13 play well-established roles in exacerbating allergic airway disease, mainly through their activation of effector cells and subsequent responses (Nakajima and Takatsu, 2007; Gour and Wills-Karp, 2015). Eotaxin is a potent chemoattractant for eosinophils and an important cell type in allergic airway pathogenesis (Conroy and Williams, 2001). Furthermore, IL-33 is a cytokine produced by cells of the lung that plays an important role in type-2 immunity and allergic inflammation (Chan et al., 2019). Given the significant influence of these cytokines in airway responses, a custom multiplex was developed to measure these cytokines in the lungs of mice repeatedly exposed to each yeast. Surprisingly, only IL-4 and IL-5 were elevated one day following repeated *C. neoformans* exposure compared to PBS control (**Figure 7**). There were slight, but nonsignificant, increases in the levels of IL-13, Eotaxin, and IL-33 one day post final exposure to either yeast compared to control. By 21 days post final exposure, there were no significant differences in the cytokine response of mice exposed to yeast or PBS control, suggesting that the cytokine response occurs during exposure and resolves by 21 days post final exposure.

## 4 Discussion

Allergic airway diseases such as asthma are a growing concern in industrialized nations like the United States (Barnes, 2004) and environmental microbial exposures have been shown to influence these diseases (Black et al., 2000). The adverse human health effects associated with environmental fungal exposures have heightened public awareness, and the development of modern methods of fungal detection in indoor environments have highlighted the need to study yeasts (Dotterud et al., 1995; Park et al., 2008; Dannemiller et al., 2014; Dannemiller et al., 2016; Rush et al., 2021). To the authors’ knowledge, this is the first *in vivo* study examining the pulmonary immune response to the environmental yeast species, *Vishniacozyma victoriae*. This is an important first study given the frequent and abundant detection of this species in indoor environments and the use of this yeast in commercial sectors. This study sought to compare the pulmonary immunological impact following repeated exposure to *V. victoriae* and *C. neoformans*. The doses (10^4^ and 10^6^ cells) chosen for this study align with concentrations identified from environmental assessments (Levitz, 1991; Rush et al., 2021). Based on preliminary epidemiological findings, it was hypothesized that *V. victoriae* would initiate a different pulmonary immune response compared to *C. neoformans*.

Pulmonary *C. neoformans* exposures exacerbate allergic airway responses (Goldman et al., 2006; Campuzano and Wormley, 2018). In this study, repeated exposure to 10^4^ *C. neoformans* cells elicited a mixed response including an influx of neutrophils and granuloma formation with macrophages and multinucleated giant cells by 21 days post final exposure. This response aligns with other studies examining this yeast that found granuloma formation following pulmonary *C. neoformans* exposure (Goldman et al., 2006). Unexpectedly, mice repeatedly exposed to 10^4^ *C. neoformans* cells had decreased total CD45^+^ cells and macrophages in the BALF 1 day post final exposure compared to PBS-exposed controls. Given that macrophages are the most abundant immune cell in the lung, this response may be due to the ability of *C. neoformans* to downregulate macrophages (Siegemund and Alber, 2008). In addition, activated macrophages following *C. neoformans* exposure may have transitioned to a “foam cell” type within granulomas and would therefore be more difficult to remove with lavage (Normile et al., 2020), which could also explain the decrease in the macrophage population. While not assessed in these studies, *C. neoformans* has been reported to initiate polarization of macrophages towards an M2 phenotype (Eastman et al., 2015). These cells are known to promote allergic airway responses (Saradna et al., 2018) and should be further investigated following future *Cryptococcus* yeast exposure studies.

Repeated exposure to *C. neoformans* resulted in an increase in both CD11b^+^ and CD103^+^ DCs and total, CD4^+^, and CD8^+^ T cells. As CD11b^+^ and CD103^+^ DCs present antigen to CD4^+^ and CD8^+^ T cells, respectively, the increase in these populations agree with results from other studies that show *C. neoformans* exposure upregulates dendritic cell activity (Wozniak et al., 2006; Siegemund and Alber, 2008; Wozniak, 2018). Overall, repeated exposure to an immunogenic dose of 10^6^ *V. victoriae* cells initiated an increased myeloid response 1 day post final exposure whereas exposure to 10^4^ *C. neoformans* cells initiated an increased myeloid response by 21 days post final exposure. This result may be due to the presence of beta glucans in the cell wall of *V. victoriae* cells, which are known to stimulate innate immune responses *via* Dectin-1 (Goodridge et al., 2009; Camilli et al., 2018). However, unlike *V. victoriae*, *C. neoformans* is able to grow at body temperature (**Supplemental Figure 2A**) and has a capsule that hides the beta glucans (**Supplemental Figure 1A**) that serves as an immune evasion mechanism. The delayed myeloid response may be in response to *in vivo* replication of *C. neoformans* and renewed presence of beta glucans present in the yeast cell wall. Dectin-1 is a pattern recognition receptor highly expressed on dendritic cells (Goodridge et al., 2009) and when stimulated, initiates dendritic cells to produce inflammatory cytokines that are essential for anti-fungal immunity (Elder et al., 2017). This is consistent with the observation that dendritic cells increased 1 day post repeated exposure to 10^6^ cells of *V. victoriae* and 21 days following repeated exposure to 10^4^ *C. neoformans* cells.

Repeated exposure to 10^4^ *V. victoriae* cells resulted in very little inflammation, airway remodeling, or cellular response (data not shown). This is not unexpected as *V. victoriae* lacks a capsule (**Supplementary Figure 1B**) and has not been previously determined to be pathogenic given its inability to replicate at body temperature (**Supplementary Figure 2B**) (Montes et al., 1999; de Menezes et al., 2019; Lutz et al., 2020; Rusinova-Videva et al., 2022). Following this observation and given that repeated exposure to 10^6^ *V. victoriae* cells resulted in a stronger response, the immunogenic dose of 10^6^ *V. victoriae* was chosen to further characterize the pulmonary immune response following exposure. The authors recognize that the comparison between the yeasts using the same dose would have been ideal; however, it was important that the selected dose initiate an immune response substantial enough to characterize mechanistically, regardless of the type of immune response. Increasing the dose of *C. neoformans* to mirror that of the increased *V. victoriae* dose could have resulted in infection and consequently, mortality. This was not the intended response of *C. neoformans* exposures for this study, but only to induce airway inflammation; therefore, the *C. neoformans* dose for exposure remained at 10^4^ cells.

Although repeated exposure to *C. neoformans* initiated a strong lymphoid response including increased B cells, total T, CD4^+^ T and CD8^+^ T cells, only total and CD4^+^ T cells were increased compared to PBS-exposed controls by 21 days post final exposure to the immunogenic dose of *V. victoriae* (10^6^ cells). This finding aligns with the increase in CD11b^+^ DCs resulting from *V. victoriae* exposure, as CD11b^+^ DCs present antigen to CD4^+^ T cells (Balan et al., 2019). The increase in CD11b^+^ DC activity and numbers of CD4^+^ T cells should be further investigated as allergen-specific CD4^+^ T cells are known drivers of allergic pulmonary responses, which may implicate the contribution of *V. victoriae* on allergic airway diseases such as asthma (Ling and Luster, 2016; Muehling et al., 2017; Jeong and Lee, 2021).

*C. neoformans* is able to persist in the lung and propagate *in vivo* (Goldman et al., 2000; Kozubowski and Heitman, 2012). The polysaccharide capsule surrounding *C. neoformans* cells is a known immune evasion mechanism (Campo et al., 2006); therefore, detectable yeast cells in the lung 21 days after final exposure was not surprising. The cellular immune responses in the BALF of mice repeatedly exposed to 10^4^ *C. neoformans* cells persisted over time as these cellular populations continued to increase from 1 day to 21 days post final exposure. By 21 days post final exposure, the cellular immune response following repeated *V. victoriae* exposure (10^6^ cells) started to resolve, unlike the response to 10^4^ *C. neoformans* cells. This observation is supported by the lack of capsule surrounding *V. victoriae* (**Supplementary Figure 1B**) and the hypothesized detection and clearance of *V. victoriae* by the pulmonary immune cells. Consequently, the detection of engulfed *V. victoriae* cells in the lungs of exposed mice 21 days after the final exposure was unanticipated. This is of particular interest given the detection of *V. victoriae* in indoor environments and increasing use of this yeast in commercial settings (Lutz et al., 2020; Rusinova-Videva et al., 2022). Future studies should focus on the *V. victoriae* cell phagocytosis, digestion, and subsequent clearance by alveolar macrophages to better understand the temporal mechanisms underlying this process. Preliminary analyses conducted in this study suggest *V. victoriae* does not produce a capsule under optimal laboratory growth conditions or following repeated *in vivo* exposures. *Vishniacozyma victoraie* stains differ in regard to the presence of absence of a capsule (de Garcia et al., 2012). Examining the lack of a capsule formation by this yeast and the potential for capsule formation under certain conditions warrant future study (Vitale et al., 2012; Jang et al., 2022). Lastly, three PBS-exposed mice were reported to have GMS^+^ histiocyte granules. Given that these mice were not exposed to yeast, it is likely that these granules were either artifact or GMS^+^ stained blood cells, which has been previously reported (Adhya, 2019).

Oropharyngeal aspiration is a commonly utilized technique for reproducible pulmonary exposure (Kinaret et al., 2017). However, the isoflurane anesthesia and aspiration process has been shown to increase damage-associated molecular patterns (DAMPs) and subsequent inflammation in the lung following exposure (Crișan et al., 2016; Roh and Sohn, 2018). As such, mild inflammation and mucous production were observed in the lungs of control mice 1 day following repeated PBS aspiration. Additionally, inflammation scores were elevated in some of the PBS-exposed groups, which is consistent with the induction of DAMPs following aspiration (Tolle and Standiford, 2013). Cellular populations such as total CD45^+^ cells and NK cells were elevated in all groups at 1-day post final exposure. By 21 days post final exposure, these populations had significantly decreased in the PBS-exposed group. This decrease in cellular infiltrates suggests that the observed inflammatory response in the PBS-exposed group may be a result of the aspiration process itself. To better understand the influence of the aspiration process on the host response, future studies should include a naive group of mice that do not undergo aspiration. In addition, other methods of exposure may better mimic a natural route of exposure, such as utilization of an inhalation chamber. Although studies that use an acoustic generator system for the aerosolization of fungal particles exist (Buskirk et al., 2014; Nayak et al., 2018; Croston et al., 2020), the morphology and structure of yeasts may make these cells more difficult to aerosolize without compromising the immunogenic profile.

Previous reports suggested that *V. victoriae* may influence the pulmonary immune response differently than pathogenic *C. neoformans*, and that exposure to *V. victoriae* may protect against allergic airway diseases (Dannemiller et al., 2016; Rush et al., 2021). The results of the current study suggest that *V. victoriae* induced a unique pulmonary immune response compared to *C. neoformans*; however, it cannot be concluded that *V. victoriae* is protective. Exposure to a single yeast species, rather than exposure to a diversified test article, could partially account for the lack of a protective effect on the pulmonary immune response. Natural exposures contain a variety of fungal species (Adams et al., 2013; Behbod et al., 2015), and it is possible that the association between *V. victoriae* and asthma may be due to *V. victoriae* occupying an environmental niche, thus keeping an allergic fungal species from propagating. Alternatively, *V. victoriae* may require interactions with secondary metabolites from other fungal species in order to elicit a protective effect (Sheridan et al., 2015).

The lack of pronounced cytokine response following exposure to each yeast was unanticipated given that cytokines play an important role in early detection and subsequent clearance of *Cryptococcus* (Campuzano and Wormley, 2018). Moreover, the lack of a strong eosinophil response compared to PBS-exposed controls was also unexpected, although an increased trend following exposure to 10^6^ *V. victoriae* cells may be appreciated. Given that samples were collected and analyzed at two timepoints, 1 day and 21 days post final exposure, it is possible that these responses are occurring at other timepoints and are therefore being missed. Future studies focused on examining the impact of these species in allergic airway disease models at various timepoints, including mixed fungal exposures that more closely mimic natural exposures in indoor environments, are warranted. Furthermore, measuring pulmonary function would also help to determine the impact of yeast exposure on respiratory health. Additionally, given that responses to the yeast are still detectable 21 days post final exposure, longer recovery timepoints may prove insightful.

In conclusion, *V. victoriae* and *C. neoformans* persisted in the lungs of mice following repeated exposure, and *C. neoformans* initiated a mixed myeloid and lymphoid response including granuloma formation that worsened over time. In contrast, repeated exposure to *V. victoriae* initiated a CD4^+^ T cell-driven lymphocytic response that began to resolve by 21 days post final exposure. Given the frequent detection, prevalence and use of *V. victoriae* in commercial settings, it is imperative that future studies further explore environmental yeast species and their impacts to continue to address this knowledge gap. The results of this study show that repeated exposure to Basidiomycota yeast species resulted in airway inflammation through different mechanisms. The unique inflammatory response elicited by *V. victoriae* further highlights the need for continued study. By examining the immune response to frequently detected species such as *V. victoriae*, the role of Basidiomycota yeast species in allergic airway disease can be better understood.

## Data availability statement

The original contributions presented in the study are included in the article/**Supplementary Material**. Further inquiries can be directed to the corresponding author.

## Ethics statement

The animal study was reviewed and approved by West Virginia University and CDC-Morgantown Animal Care and Use Committees.

## Author contributions

RR, BG, KD, and TC conceived the overall project. RR cultivated the fungal organisms with input from KD. RR, CB, and TC performed the fungal exposures. AL analyzed mucicarmine stained slides and conducted the growth curve studies. RR, CB, AL, and TC performed euthanasia and tissue necropsy. RR performed flow cytometry panel design and analysis with assistance from CB. RR performed cytokine analyses. RR and TC wrote the manuscript with input from all the authors. All authors contributed to the article and approved the submitted version.
