# Corrigendum: Persisting *Cryptococcus* yeast species *Vishniacozyma victoriae* and *Cryptococcus neoformans* elicit unique airway inflammation in mice following repeated exposure

**DOI:** 10.3389/fcimb.2024.1381148

**Published:** 2024-02-16

**Authors:** Rachael E. Rush, Catherine B. Blackwood, Angela R. Lemons, Karen C. Dannemiller, Brett J. Green, Tara L. Croston

**Affiliations:** ^1^Department of Microbiology, Immunology and Cell Biology, West Virginia University, Morgantown, WV, United States; ^2^Health Effects Laboratory Division, National Institute for Occupational Safety and Health, Centers for Disease Control and Prevention, Morgantown, WV, United States; ^3^Department of Civil, Environmental & Geodetic Engineering, College of Engineering, Ohio State University, Columbus, OH, United States; ^4^Division of Environmental Health Sciences, College of Public Health, Ohio State University, Columbus, OH, United States

**Keywords:** yeast, fungi, inflammation, allergic disease, Vishniacozyma victoriae, Cryptococcus neoformans, exposure

**Error in Author List**

In the published article, there was an error in the author list, and author Karen C. Dannemiller was erroneously excluded. The corrected author list appears below.

Rachael E. Rush^1,2^, Catherine B. Blackwood^2^, Angela R. Lemons^2^, Karen C. Dannemiller^3,4^, Brett J. Green^2^ and Tara L. Croston^2^*

In addition, the **Author Contributions** section has been updated:

“RR, BG, KD, and TC conceived the overall project. RR cultivated the fungal organisms with input from KD. RR, CB, and TC performed the fungal exposures. AL analyzed mucicarmine stained slides and conducted the growth curve studies. RR, CB, AL, and TC performed euthanasia and tissue necropsy. RR performed flow cytometry panel design and analysis with assistance from CB. RR performed cytokine analyses. RR and TC wrote the manuscript with input from all the authors. All authors contributed to the article and approved the submitted version.”

The authors apologize for this error and state that this does not change the scientific conclusions of the article in any way. The original article has been updated.

