# Supplementary material for: Persisting Cryptococcus yeast species Vishniacozyma victoriae and Cryptococcus neoformans elicit unique airway inflammation in mice following repeated exposure
Source: Front Cell Infect Microbiol. 2023 Feb 14;13:1067475. doi: 10.3389/fcimb.2023.1067475 (PMC9971225; doi:10.3389/fcimb.2023.1067475)
Supplement: Supplementary file 1 [file DataSheet_1.pdf]

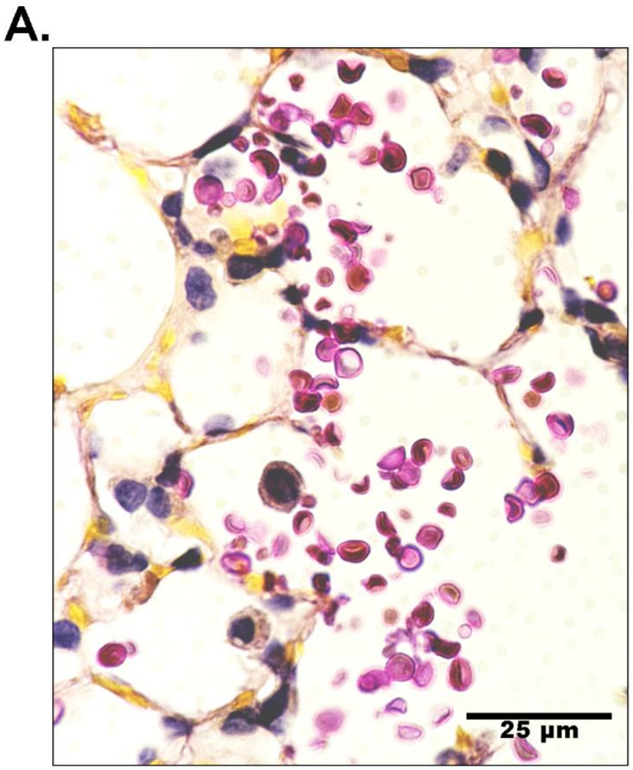

Supplemental Figure 1. Mucicarmine staining of *V. victoriae* and *C. neoformans* cells in murine lungs. *C. neoformans* cells stain magenta (A) while *V. victoriae* cells stain dark purple (B), indicating the presence of a polysaccharide capsule surrounding *C. neoformans* cells but not *V. victoriae* cells. Magnification=100X (oil immersion).

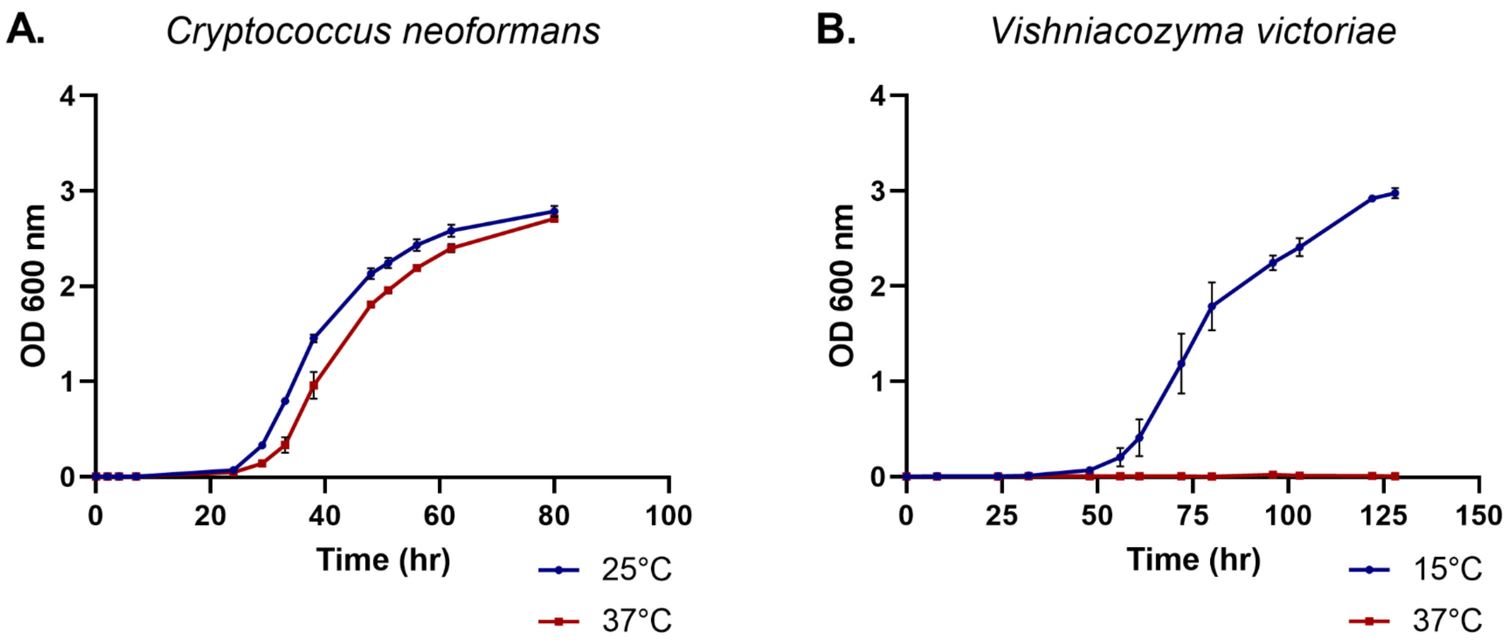

Supplemental Figure 2. Growth of *V. victoriae* and *C. neoformans* at optimal temperature versus 37°C. *C. neoformans* grew well at both 25°C (A, blue) and 37°C (A, red), while *V. victoriae* grew optimally at 15°C (B, blue) but not at all at 37°C (B, red). N=2 per growth condition, error bars represent standard deviation.

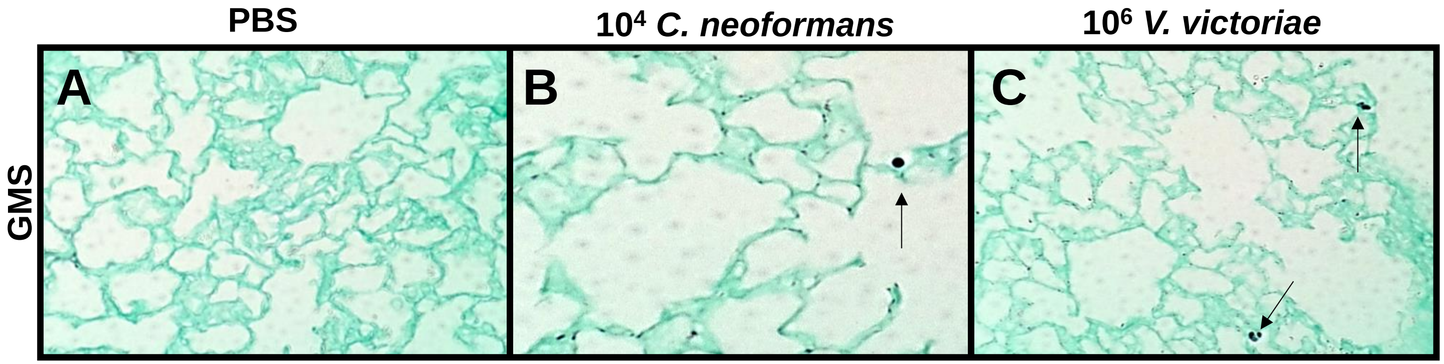

Supplemental Figure 3: Deposition of yeast cells in lung following a single oropharyngeal aspiration exposure. Representative micrographs of GMS-stained sections of mouse lungs approximately one hour following one exposure to PBS (left), 10^4 *C. neoformans* cells (middle), or 10^6 *V. victoriae* cells (right). Yeast cells are indicated by arrows. 400x magnification.

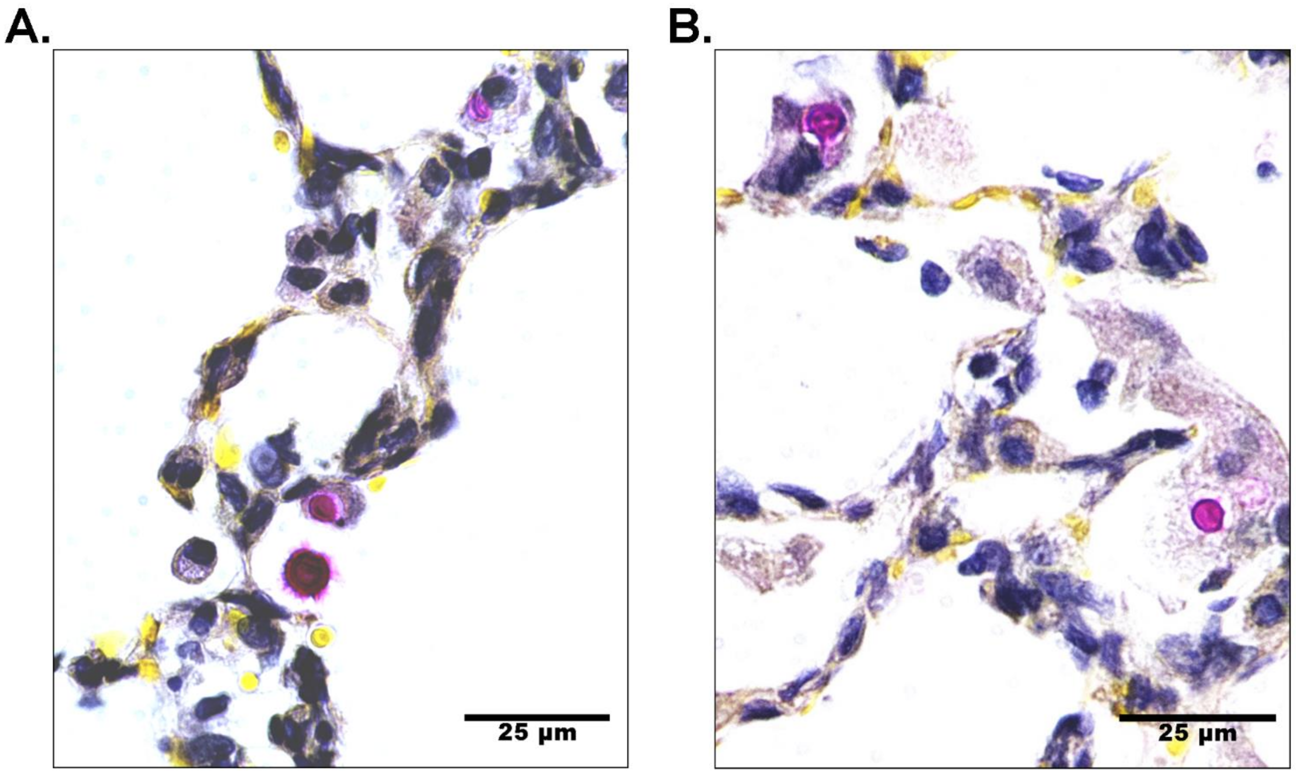

Supplemental Figure 4. Mucicarmine staining of *C. neoformans* cells in murine lungs following repeated oropharyngeal aspiration exposure. *C. neoformans* cells stained magenta in lung tissue 1-day (A) and 21-days (B) following repeated exposure to 10^4 cells. Magnification=100X (oil immersion).

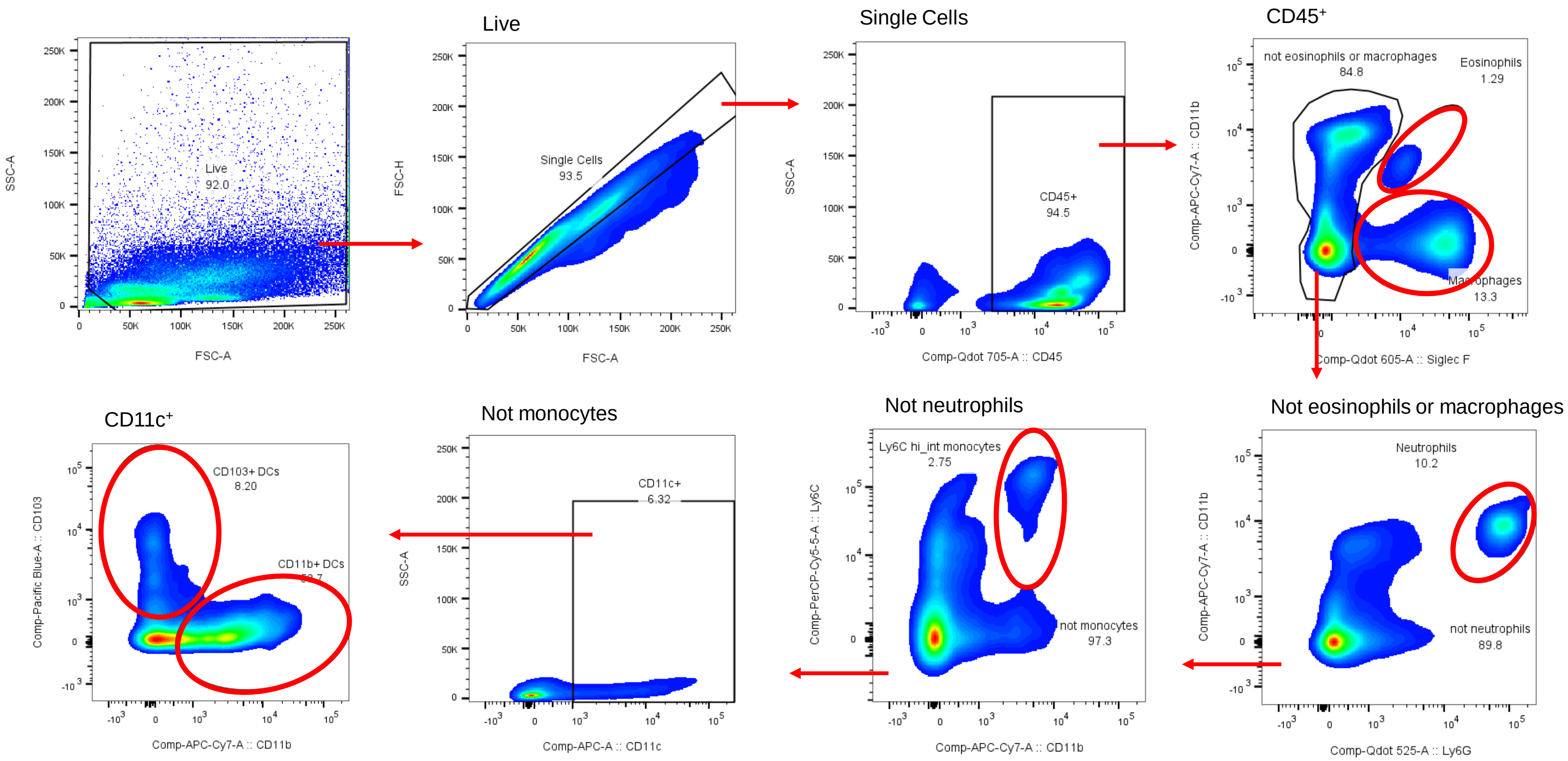

Supplemental Figure 5A: Flow cytometry gating strategy (myeloid panel). Representative dot plots showing gating strategy for myeloid cell populations of interest. Live cells were gated based on FSC-A and SSC-A, then single cells were gated from FSC-A and FSC-H. CD45^{+} Cells were selected and gated on Siglec F and CD11b to isolate eosinophils and macrophages. Non-eosinophils and non-macrophages were gated on Ly6G and Cd11b to isolate neutrophils. The non-neutrophil population was then gated on CD11b and Ly6C to isolate Ly6C$^{hi/med}$ monocytes. Non-monocytes were gated on CD11c to isolate dendritic cells that further gated on CD11b and CD103 for specific CD11b^{+} and CD103^{+} DC populations.

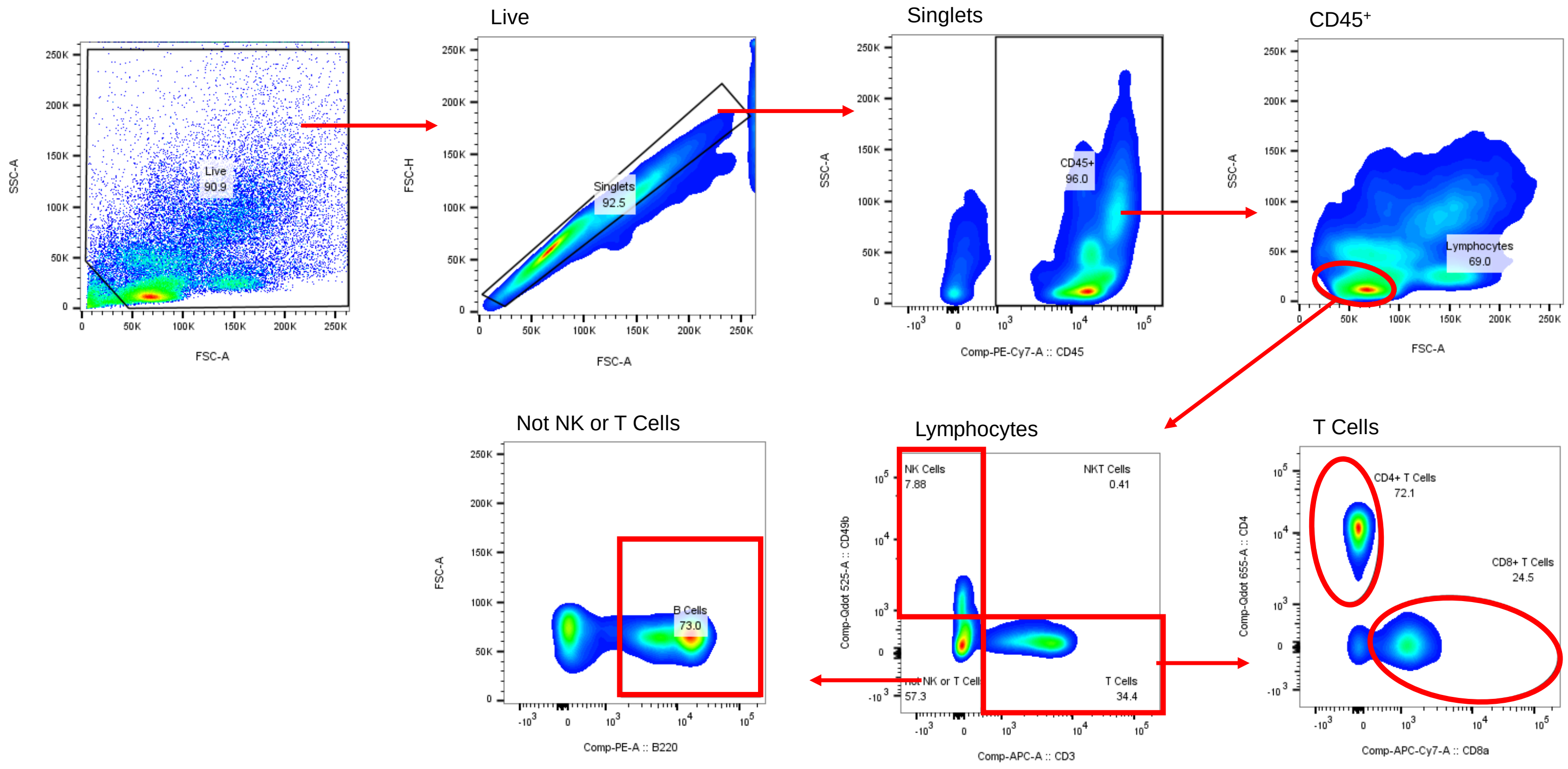

Supplemental Figure 5B: Flow cytometry gating strategy (lymphoid panel). Representative dot plots showing gating strategy for lymphoid cell populations of interest. Live cells were gated based on FSC-A and SSC-A, then single cells were gated from FSC-A and FSC-H. CD45$^+$ Cells were selected then lymphocytes were gated out based on size. Lymphocytes were then gated on CD49b and CD3 to isolate NK and T Cells, respectively. T cells were further gated on CD8a and CD4 to isolate CD8$^+$ and CD4$^+$ T cell subpopulations. Non-NK or non-T Cells were gated against B220 to isolate B cells.

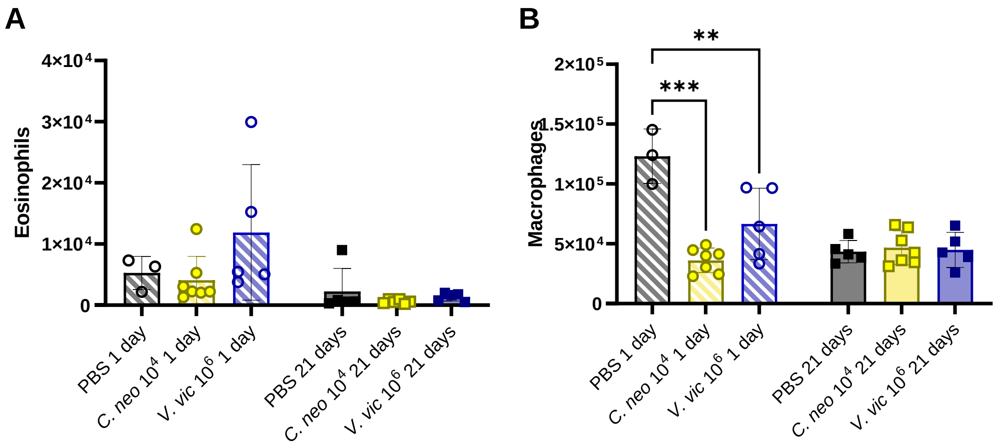

Supplemental Figure 6: Cell populations in BALF following repeated yeast exposure. Cell populations in BALF following repeated exposure to PBS (black), 10^4 *C. neoformans* cells (yellow) or 10^6 *V. victoriae* cells (blue) at 1 day post final exposure (circles, crossed bars, left) or 21 days post final exposure (squares, solid bars, right). A) Eosinophils, B) Macrophages. n=3-7 per group, *P<0.05, **P < 0.01, ***P < 0.001. P values were determined by ordinary one-way ANOVA with Tukey's multiple comparisons test comparing each exposure together for the same timepoint.

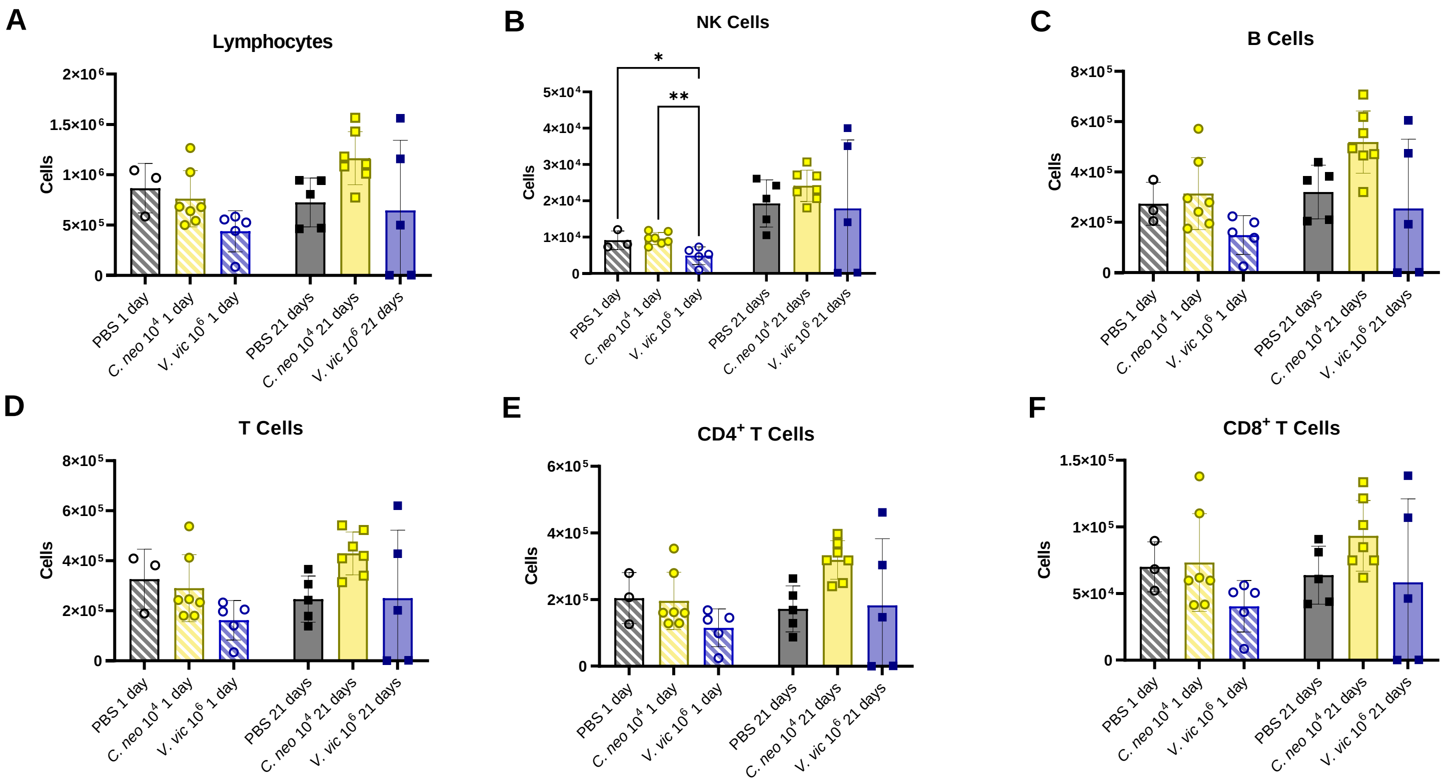

Supplemental Figure 7: Lymphoid cell populations in BAL-depleted Lung following repeated yeast exposure. Cell populations in BAL-depleted lung following repeated exposure to PBS (black), 10^4 *C. neoformans* cells (yellow) or 10^6 *V. victoriae* cells (blue) at 1 day post final exposure (circles, crossed bars, left) or 21 days post final exposure (squares, solid bars, right). A) Lymphocytes, B) NK Cells, C) B Cells, D) T Cells, E) $CD4^+$ T Cells, F) $CD8^+$ T Cells. n=3-7 per group, $^*P<0.05$, $^{**}P < 0.01$. P values were determined by ordinary one-way ANOVA with Tukey's multiple comparisons test comparing each exposure together for the same timepoint.

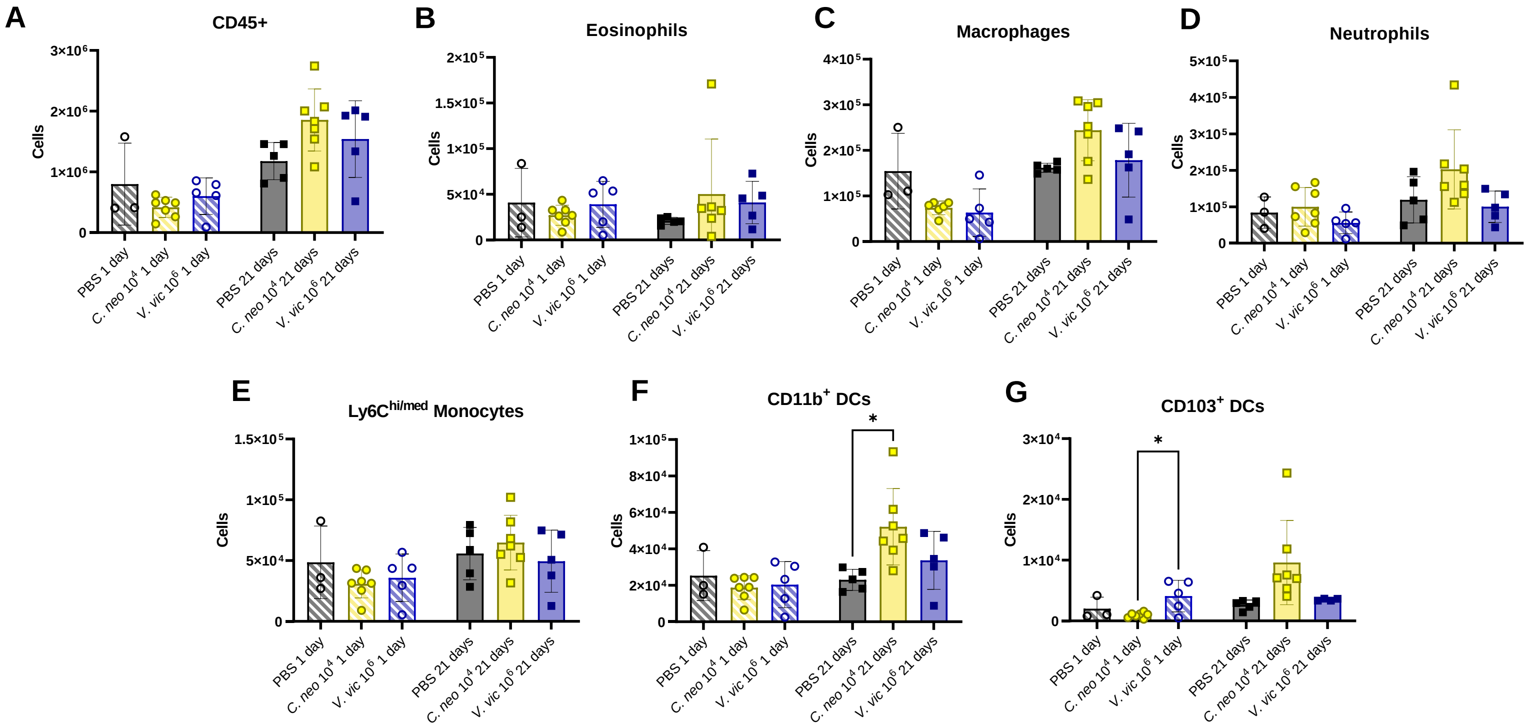

Supplemental Figure 8: Myeloid cell populations in BAL-depleted Lung following repeated yeast exposure. Cell populations in BAL-depleted lung following repeated exposure to PBS (black), 10^4 *C. neoformans* cells (yellow) or 10^6 *V. victoriae* cells (blue) at 1 day post final exposure (circles, crossed bars, left) or 21 days post final exposure (squares, filled bars, right). A) $CD45^+$, B) Eosinophils, C) Macrophages, D) Neutrophils, E) $Ly6C^{hi/med}$ Monocytes, F) $CD11b^+$ Dendritic Cells, G) $CD103^+$ Dendritic Cells. n=3-7 per group, *$P<0.05$, **$P < 0.01$. P values were determined by ordinary one-way ANOVA with Tukey's multiple comparisons test comparing each exposure together for the same timepoint.

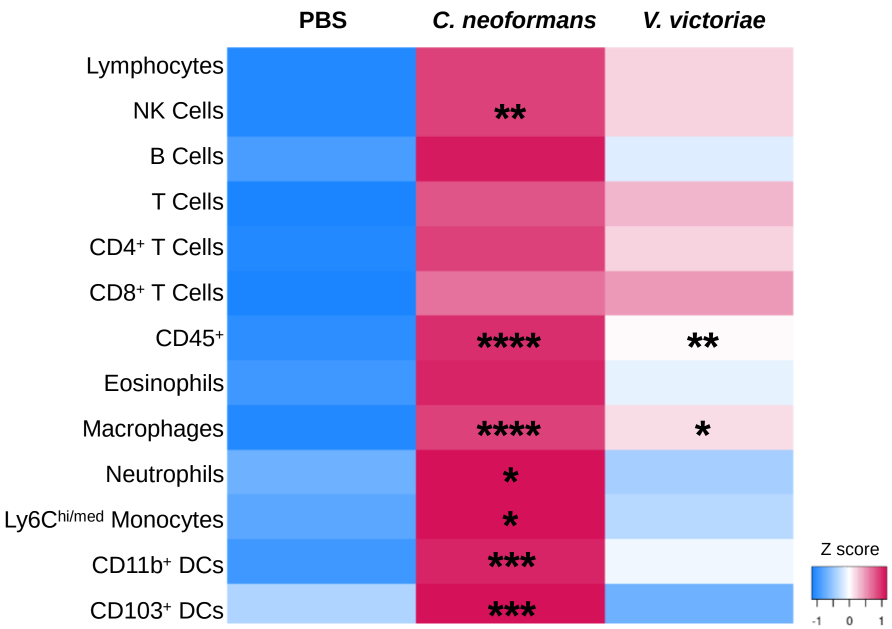

Supplemental Figure 9: Changes in BAL-depleted Lung cell population quantifications from 1 day to 21 days post final exposure. Mean cell quantifications in lung following repeated exposure to PBS, 10^4 *C. neoformans* cells or 10^6 *V. victoriae* cells at 1 day post final exposure compared to 21 days post final exposure. n=3-7 per group. Values used for this heatmap were obtained by calculating the average quantities at 21 days post final exposure and subtracting the average quantities at 1 day post final exposure. A positive Z score (pink) indicates that the quantification of cells increased at 21 days post final exposure compared to 1 day post final exposure. Negative Z scores (blue) indicate that the cell quantifications decreased by 21 days post final exposure compared to 1 day post final exposure. Asterisks overlaying the heatmap indicate P values comparing the quantifications of each time point for the same exposure. *P<0.05, **P < 0.01, ***P < 0.001, ****P < 0.0001. P values were determined by ordinary one-way ANOVA with Sidak's multiple comparisons test comparing the same exposure at 1 day post final exposure and 21 days post final exposure.

Supplemental Table 1: Flow Cytometry Antibodies and Reagents

Lymphoid Panel:					
Marker	*Species*	*Fluorochrome*	*Clone*	*Company*	*Catalog Number*
CD45	Rat anti-mouse	PE Cy 7	I3:2.3	Southern Biotech	166017
CD49b	Rat anti-mouse	EF 506	DX5	Invitrogen	69-5971-82
CD3	Rat anti-mouse	APC	17A2	Invitrogen	17-0032-82
CD4	Rat anti-mouse	BV 650	RM4-5	Biolegend	100545
CD8a	Rat anti-mouse	APC Cy 7	53-6.7	BD	561967
B220	Rat anti-mouse	PE	RA3-6B2	BD	553090
**Myeloid Panel:**					
Marker	*Species*	*Fluorochrome*	*Clone*	*Company*	*Catalog Number*
CD45	anti-mouse	BV 711	30-F11	Biolegend	103147
CD11b	Rat anti-mouse/human	APC Cy 7	M1/70	BD	561039
Siglec F	Rat anti-mouse	BV 605	E50-2440	BD	740388
Ly6G	Rat anti-mouse	BV 510	1A8	BD	740157
Ly6C	Rat anti-mouse	Per CP Cy 5.5	HK1.4	Invitrogen	45-5932-80
CD11c	anti-mouse	APC	N418	Biolegend	117310
CD103	Rat anti-mouse	BV 421	M290	BD	562771

Reagents:		
Name	*Company*	*Catalog Number*
DMEM, high glucose, pyruvate	Gibco	11995-040
Fetal Bovine Serum	Gibco	1600-044
Collagenase/Dispase	Sigma Aldrich	11097113001
Deoxyribonuclease I	Alfa Aesar	J61061
10x RBC Lysis Buffer	Invitrogen	00-4300-54
PBS 1x	HyClone	SH30256.02
EDTA 0.5 M, pH 8.0	Affymetrix	15684
Rat Serum	Sigma Aldrich	S7648
CD16/CD32 Rat anti-mouse unlabeled Clone 2.4G2	BD	553141
Cytofix Fixation Buffer	BD	554655
Sphero AccuCount Blank Particles	Spherotech Inc	ACBP-50-10
Ultra Comp eBeads	Invitrogen	01-2222-42
Rabbit anti-Mouse IgM Secondary Antibody, AP	Invitrogen	31333
IgG Fc Goat anti-Mouse, AP	Invitrogen	31325
Goat Anti-Mouse IgE-AP	Southern Biotech	1110-04
Thermo Sceintific Pierce PNPP Substrate Kit	Invitrogen	37620

Supplemental Table 1: Flow Cytometry Antibodies and Reagents. Antibody and reagent names, manufacturers and catalog numbers.

Supplemental Table 2 – Repeated Exposure Histology Scores

Group:	Specimen	H&E: Mononuclear Inflammation: Perivascular	H&E: Mononuclear Inflammation: Peribronchial	H&E: Mononuclear Inflammation: Parenchymal	H&E: Granulomatous	H&E: Alveolar Histiocytes	H&E: Inflammation Score	PAS: Goblet cells	GMS: Yeast	GMS: Histiocyte granules
PBS 1 day	F52	0	0	0	0	0	0	1	0	0
	F53	1	0	0	0	1	2	1	0	1
	F54	1	0	0	0	1	2	1	0	1
C. neo 10^4 1 day	F45	1	1	0	0	1	3	2	1	1
	F46	1	1	0	0	0	2	0	0	1
	F47	1 mixed	2 mixed	2	2	2	6	2	2	2
V. vic 10^4 1 day	F61	0	0	0	0	0	0	1	0	1
	F62	0	0	0	0	0	0	0	0	1
	F63	1	1	0	0	0	2	0	0	1
V. vic 10^6 1 day	F70	2	2	0	0	2	6	2	0	1
	F71	2	1	0	0	1	4	2	0	2
	F72	1	2	0	0	1	4	3	0	2
PBS 21 days	F06	0	0	0	0	0	0	1	0	0
	F07	0	0	0	0	0	0	1	0	0
	F08	1	1	0	0	0	2	0	0	1
C. neo 10^4 21 days	F16	2 mixed	2 mixed	0	2	2	4	2	0	1
	F17	2	2	2	3	0	9	4	2	2
	F18	2	2	1	1	2	8	3	1	2
V. vic 10^4 21 days	F26	0	0	0	0	0	0	0	0	1
	F27	0	0	0	0	0	0	1	0	1
	F28	0	0	0	0	0	0	1	0	0
V. vic 10^6 21 days	F35	1	0	0	0	1	2	0	0	2
	F36	1	1	0	0	1	3	2	0	1
	F37	1	1	0	0	0	2	2	0	2

*mixed = neutrophils also present
1 day = tissue collected one day after final exposure
21 days = tissue collected 21 days after final exposure

Supplemental Table 2: Repeated Exposure Histology Scores. Provided histology scores from blinded histopathologist examining sectioned and stained lung tissue from mice repeatedly exposed to yeast or PBS control. H&E = hematoxylin and eosin stain, PAS = Periodic acid shift stain, and GMS = Grocott's methenamine silver stain.
